# ‘How Are We Going to Learn This Profession?’: A Multi-Stakeholder Qualitative Study of Educational Disruption Across Pandemic and Earthquake Crises

**DOI:** 10.3390/healthcare14213772

**Published:** 2026-11-04

**Authors:** Beril Nisa Yaşar, Sema Çifçi, Sibel İçke

**Affiliations:** 1 Midwifery Department, Faculty of Health Sciences, Mardin Artuklu University, Mardin 47200, Türkiye; sibelicke@artuklu.edu.tr; 2 Nursing Department, Faculty of Health Sciences, Mardin Artuklu University, Mardin 47200, Türkiye; semacifci@artuklu.edu.tr

**Keywords:** nursing education, midwifery education, clinical learning, professional competence, online education, hybrid education, COVID-19, earthquake, qualitative research

## Abstract

**Highlights:**

**What are the main findings?**

- Pandemic-related interruptions to face-to-face education were associated with perceived losses in experiential and clinical learning, whereas post-earthquake changes primarily affected theoretical course delivery.
- Students, faculty members, and healthcare professionals expressed related concerns about clinical learning and professional preparedness from different educational and clinical perspectives.

**What are the implications of the main findings?**

- Crisis preparedness should protect supervised experiential learning rather than focus solely on continuity of theoretical course delivery.
- Adaptable clinical learning pathways, appropriate assessment, technological support, and educational–clinical collaboration should be incorporated into contingency planning.

**Abstract:**

**Background/Objectives:** Crisis-related disruptions pose particular challenges for nursing and midwifery education because maintaining educational continuity also requires preserving experiential and clinical learning. The COVID-19 pandemic and the February 2023 earthquakes in Türkiye prompted changes in educational delivery. While the pandemic involved prolonged interruptions to face-to-face and clinical education, post-earthquake arrangements at the study institution primarily affected theoretical courses, with clinical and practical training continuing face-to-face. This study aimed to explore the experiences and perspectives of nursing and midwifery students, faculty members, and healthcare professionals regarding online and hybrid education implemented during the COVID-19 pandemic and the 2023 earthquakes in Türkiye, including its perceived implications for clinical learning and professional competence. **Methods:** This qualitative study employed a descriptive phenomenological design. Criterion purposive sampling was used to recruit 48 participants, including 20 senior nursing and midwifery students, 12 faculty members, and 16 nurses and midwives working with students in clinical settings. Data were collected through 11 face-to-face focus group interviews conducted between 15 and 26 May 2023. Interviews were audio-recorded, transcribed verbatim, and analyzed using Colaizzi’s phenomenological analysis method. Two researchers independently analyzed the data, and credibility was further supported through external expert review and member checking. **Results:** Four interconnected themes emerged: (1) disrupted learning and unequal participation, (2) loss of experiential and clinical learning, (3) from educational disruption to professional competence concerns, and (4) reconsidering educational strategies during crises. Participants described technological and contextual barriers to educational participation across the two crises. Disruptions to laboratory and clinical learning, including reduced opportunities to develop and practice clinical skills, were primarily associated with the prolonged interruption of face-to-face education during the COVID-19 pandemic, whereas post-earthquake changes at the study institution mainly concerned theoretical course delivery. Students reported uncertainty about their clinical abilities and preparedness for professional practice, while faculty members and healthcare professionals described similar concerns from educational and clinical perspectives. Despite these challenges, participants recognized the value of online education for selected theoretical content and reported gains in digital skills. They emphasized preserving practice-based learning, strengthening technological and financial support, and involving relevant stakeholders in crisis-related educational decisions. **Conclusions:** Participants associated pandemic-related interruptions to experiential and clinical learning with concerns about professional preparedness, whereas their reflections on post-earthquake educational arrangements highlighted the importance of adapting theoretical course delivery to the circumstances of the crisis while maintaining face-to-face clinical training. The convergence of perspectives across students, faculty members, and healthcare professionals highlights the importance of protecting supervised experiential learning while maintaining educational continuity. Crisis preparedness in nursing and midwifery education should therefore incorporate adaptable educational models, contingency plans for clinical learning, appropriate assessment strategies, technological support, and collaboration among educational and clinical stakeholders.

## 1. Introduction

Health profession education is particularly vulnerable to disruptions caused by large-scale crises such as pandemics and natural disasters because these events may interrupt not only classroom-based teaching but also clinical placements and practice-based learning opportunities [1]. In practice-oriented disciplines such as nursing and midwifery, professional preparation requires more than the acquisition of theoretical knowledge; clinical experience, hands-on practice, communication skills, confidence, and opportunities to develop professional competencies are integral to readiness for professional practice [2]. A recent systematic review of 93 studies from 32 countries demonstrated that readiness for professional practice is shaped by interconnected individual, educational, and contextual factors, with clinical placements, curriculum design, mentorship, supervision, and supportive workplace environments playing particularly important roles [2]. Disruptions to these learning opportunities may therefore have consequences beyond temporary interruptions in education. Evidence from nursing education during the COVID-19 pandemic indicates that reduced opportunities for skills practice, changes in clinical placements, uncertain practice supervision, and distance learning affected students’ confidence and perceived competence and contributed to concerns about demonstrating clinical proficiency [1]. These findings highlight the importance of examining educational disruption in health professions education not only in terms of maintaining instructional continuity but also in terms of its potential implications for students’ readiness for clinical and professional practice.

The COVID-19 pandemic prompted an unprecedented and rapid transition from conventional face-to-face education to emergency remote and online teaching across higher education institutions worldwide [3]. Although this transition enabled educational activities to continue during periods of restricted physical access, its implementation in nursing and midwifery education presented particular challenges because these programs depend substantially on experiential, laboratory, and clinical learning [4]. Evidence synthesized from nursing students and faculty members has shown that the abrupt transition to remote education was accompanied by difficulties related to technological access, student engagement, communication, assessment, faculty preparedness, and the replacement or postponement of clinical learning experiences [4]. More recent evidence suggests that online approaches can support the acquisition of some practical nursing skills when appropriately designed; however, their effectiveness varies according to the type of skill, educational strategy, and technological modality used [5]. Importantly, a recent qualitative evidence synthesis of nursing and midwifery faculty experiences concluded that clinical placements and practical skills training remain essential components of in-person education, while online approaches may be more appropriately used to complement rather than fully replace face-to-face learning [6]. Thus, the central challenge for nursing and midwifery education during crises is not simply whether education can continue remotely, but how continuity can be maintained without compromising the experiential and clinical learning required for professional preparation.

The challenges associated with educational disruption are not limited to pandemics. Türkiye is particularly vulnerable to earthquakes because of its complex tectonic setting and active fault systems, including the North Anatolian and East Anatolian faults. This persistent seismic hazard underscores the importance of disaster preparedness and educational continuity planning in higher education institutions [7].

Natural disasters can also interrupt higher education and require institutions to rapidly reorganize teaching, assessment, student support, and access to educational environments [8]. In Türkiye, the earthquakes of 6 February 2023 created a further major disruption to higher education shortly after universities had navigated the extensive educational changes associated with the COVID-19 pandemic. Following the earthquakes, the Council of Higher Education initially introduced distance education for the 2022–2023 spring semester and subsequently revised these arrangements to allow face-to-face education alongside distance education under specified conditions [9,10]. Evidence from the post-earthquake period in Türkiye has shown that students and instructors experienced challenges related to communication, institutional support, changing educational arrangements, motivation, technological access, and family circumstances [8]. For students in practice-based health professions, such successive crisis-related disruptions warrant particular attention because changes in educational delivery occur alongside the continuing need to acquire clinical skills and prepare for professional practice. Examining nursing and midwifery education across successive disruptions may therefore provide insights into educational continuity, clinical learning, and perceived preparedness for practice that cannot be captured by examining emergency remote education during the COVID-19 pandemic alone.

Existing research on crisis-related disruptions in nursing education has largely examined the experiences of students or faculty members, either separately or together, with particular attention to the transition to online education during the COVID-19 pandemic [4,11]. More recent studies have extended this focus to clinical readiness and the transition to professional practice, demonstrating concerns related to confidence, clinical and communication skills, and adaptation to the clinical environment among students and graduates whose education was affected by the pandemic [1,12,13]. Clinical educators provide an important complementary perspective because they are positioned to observe how educational experiences are reflected in students’ and graduates’ performance within real clinical environments [13]. Nevertheless, evidence integrating the perspectives of students, academic faculty, and healthcare professionals who work with students in clinical settings remains limited, particularly in the context of successive crisis-related educational disruptions. Examining these perspectives together may provide a more comprehensive understanding of how disruptions are experienced across the educational–clinical continuum, from teaching and learning to clinical participation and perceived preparedness for professional practice. Therefore, this study aimed to explore the experiences and perspectives of nursing and midwifery students, faculty members, and healthcare professionals regarding online and hybrid education implemented during the COVID-19 pandemic and the 2023 earthquakes in Türkiye, including its perceived implications for clinical learning and professional competence.

## 2. Materials and Methods

### 2.1. Study Design and Setting

This qualitative study employed a descriptive phenomenological approach to explore the lived experiences and perspectives of nursing and midwifery students, faculty members, and healthcare professionals regarding educational disruptions associated with the COVID-19 pandemic and the February 2023 earthquakes in Türkiye. A phenomenological approach was considered appropriate because the study sought to understand how participants experienced and attributed meaning to changes in educational delivery across these crisis-related disruptions [14,15].

Colaizzi’s phenomenological analysis method was used to systematically examine the meanings participants attributed to their experiences.

Although phenomenological research frequently relies on individual interviews, focus group interviews were considered appropriate because participants had experienced educational disruptions within shared academic or clinical contexts. The group discussions enabled participants to articulate their individual experiences, reflect on common circumstances, and elaborate on perspectives raised by others. During analysis, attention was given to the meanings conveyed in individual accounts rather than assuming that the perspectives expressed within each group represented a uniform or consensual experience. The study was reported in accordance with the Consolidated Criteria for Reporting Qualitative Research (COREQ) [16].

The study was conducted at the Nursing and Midwifery Departments of Mardin Artuklu University and a training and research hospital where students from these programs undertook clinical practice.

In Türkiye, undergraduate nursing and midwifery education comprises four years (eight semesters). The students included in this study began their undergraduate education in the fall semester of 2019 and completed it in 2023. Their educational experiences included several changes in delivery mode associated with the COVID-19 pandemic and the February 2023 earthquakes. Following the transition to distance education in March 2020, education was predominantly delivered remotely during the subsequent pandemic-affected semesters. Face-to-face education resumed in the spring semester of 2022. Following the February 2023 earthquakes, theoretical courses were delivered remotely until April 2023 and subsequently through a hybrid approach, whereas clinical and practical training continued face-to-face from the beginning of the spring semester. Online teaching was delivered through the university’s Learning Management System (LMS), which supported synchronous course delivery, recording of sessions, uploading of course materials, and subsequent access to recorded content. The semester-by-semester educational delivery timeline is presented in Table 1.

### 2.2. Participants and Sampling

Criterion purposive sampling was used to recruit participants who had direct experience with the educational disruptions examined in the study [17]. Three stakeholder groups were included to capture perspectives across both academic and clinical learning environments: nursing and midwifery students, faculty members, and healthcare professionals who worked with these students during clinical practice.

Eligible students were active senior nursing or midwifery students who had been enrolled during the 2019–2023 period and had experienced educational changes associated with both the COVID-19 pandemic and the 2023 earthquakes. Faculty members were eligible if they had taught practical nursing or midwifery courses for at least one semester during this period. Healthcare professionals were eligible if they were nurses or midwives who had worked with nursing or midwifery students in the clinical setting for at least one semester between 2019 and 2023. Participation was voluntary, and individuals who did not wish to participate were not included. Eligible individuals were identified according to the predefined inclusion criteria and invited directly by members of the research team. No invited individuals declined participation, and no participants withdrew after the focus group interviews had begun.

A total of 48 participants were included: 20 senior students (10 nursing and 10 midwifery students), 12 faculty members (eight from nursing and four from midwifery), and 16 healthcare professionals (eight nurses and eight midwives). Participants were organized into 11 focus groups. Nursing students participated in two groups (*n* = 4 and *n* = 6), and midwifery students in two groups (*n* = 5 and *n* = 5). Nursing faculty participated in two groups (*n* = 4 in each), whereas midwifery faculty participated in one group (*n* = 4). Nurses and midwives each participated in two focus groups of four participants.

Healthcare professionals were recruited based on their experience of working with nursing and midwifery students in clinical settings during the study period. During the focus group discussions, their accounts included observations concerning both nursing and midwifery students and newly entering healthcare staff. These observations were not systematically linked to individual students participating in the study.

### 2.3. Research Team and Reflexivity

The focus group interviews were conducted by two members of the research team (BNY and SÇ), who were also involved in the qualitative data analysis. All 11 focus group interviews were moderated by BNY, while the other researcher was responsible for recording field notes and contextual observations. These roles remained consistent throughout data collection and were not alternated between focus groups.

Both researchers were familiar with the educational and clinical contexts in which the study was conducted. Although neither researcher had taught the participating students, the researchers worked within the same institutional environment, and other pre-existing academic or professional familiarity with some participants was possible. The research team recognized that such familiarity could influence both participants’ willingness to express their views and the researchers’ interpretation of the data. To reduce the influence of individual researcher perspectives during analysis, the two researchers independently reviewed the audio recordings and transcripts and developed their initial interpretations separately. Their interpretations and emerging themes were subsequently compared; disagreements were discussed by returning to the original data, and the thematic structure was additionally reviewed by an academic expert outside the research team with expertise in qualitative and phenomenological research.

Reflexivity was addressed through ongoing discussions between the researchers during data analysis. The researchers critically examined their interpretations, discussed potential influences of their prior experiences and familiarity with the educational context, and revisited the original transcripts when differences in interpretation emerged. Although a formal, documented bracketing procedure was not undertaken before data collection, these discussions provided opportunities to identify and critically reflect on potential researcher assumptions throughout the analytical process.

### 2.4. Data Collection

Data were collected between 15 and 26 May 2023, through 11 face-to-face focus group interviews. Separate semi-structured interview guides were developed for students, faculty members, and healthcare professionals to reflect their different roles and experiences within the educational and clinical environments. The interview guides were developed by the research team based on a review of the relevant literature and were subsequently reviewed by faculty members outside the research team who had expertise in nursing and midwifery education and taught practice-based courses. Expert feedback was used to assess the relevance, clarity, and appropriateness of the questions for the respective participant groups, and the interview guides were finalized accordingly. The final guides included seven open-ended questions for students, eight for faculty members, and seven for healthcare professionals. The questions explored participants’ experiences with the different educational approaches implemented since the COVID-19 pandemic, their perceptions of these approaches, challenges encountered during online and hybrid education, suggested solutions, and the perceived reflections of educational changes on clinical practice.

Before each focus group, participants were informed about the purpose and scope of the study, the interview process, confidentiality, and the voluntary nature of participation. Interviews were scheduled at mutually appropriate times and conducted in quiet rooms at the university or hospital. With participants’ permission, all interviews were audio-recorded. During the interviews, BNY served as the moderator, while SÇ documented field notes, including participants’ non-verbal expressions, tone of voice, facial expressions, and other contextual observations.

The semi-structured format allowed participants to describe their experiences and perspectives in their own words. The order of questions was flexible, and probing questions were used when responses required clarification or further elaboration. Participants were given sufficient time to reflect on and describe their experiences. Audio recordings were transcribed verbatim and integrated with the field notes for analysis. Data saturation was assessed separately across the three main stakeholder categories: students, faculty members, and healthcare professionals. During data collection, the researchers continuously evaluated whether additional focus group discussions generated new information or perspectives relevant to the research questions within each stakeholder category. Data collection was concluded when subsequent discussions no longer yielded substantially new information. Saturation was assessed at the stakeholder-category level rather than independently for each professional subgroup; therefore, saturation was not claimed separately for the midwifery faculty subgroup, which comprised a single focus group of four participants. A total of 11 focus group interviews were conducted, with individual sessions lasting between 53 and 94 min and a combined duration of 10 h and 58 min.

The semi-structured focus group interview guides used for students, faculty members, and healthcare professionals are provided in Supplementary File S1, and the completed COREQ checklist is provided in Supplementary File S2.

### 2.5. Data Analysis

Descriptive characteristics of the participants were summarized using frequencies, percentages, and means, as appropriate. Qualitative data were analyzed using Colaizzi’s phenomenological analysis method [15]. The qualitative data were analyzed manually without the use of specialized qualitative data analysis software. Colaizzi’s method provides a systematic approach to describing lived experiences through the identification of significant statements, formulation of meanings, organization of these meanings into thematic clusters, and development of a comprehensive description of the phenomenon. The approach also emphasizes returning to participants to assess whether the resulting description adequately reflects their experiences. Audio recordings were transcribed verbatim and considered together with the field notes recorded during the focus group interviews. Two researchers (BNY and SÇ) independently listened to the audio recordings and repeatedly read the transcripts to become familiar with the participants’ accounts and gain an overall understanding of their experiences.

The analysis proceeded iteratively in accordance with Colaizzi’s approach. Significant statements directly related to participants’ experiences of online and hybrid education during the crisis periods were identified, and meanings were formulated from these statements. The formulated meanings were compared across the dataset and organized into related subthemes, which were subsequently integrated into broader themes describing the shared patterns and variations in participants’ experiences. During this process, conceptually similar and closely related themes were merged to develop the final four overarching themes. No themes were excluded from the analysis; rather, the thematic structure was refined by integrating overlapping themes while preserving the meanings represented in participants’ accounts. The two researchers conducted the initial analysis independently and then compared their interpretations, formulated meanings, subthemes, and emerging themes. When differences in interpretation occurred, the relevant transcripts and audio recordings were revisited and discussed until agreement was reached. The resulting thematic structure was further reviewed against the original data to ensure that the themes remained grounded in participants’ accounts. The focus group interviews were conducted in Turkish, and the original transcripts were analyzed in Turkish to preserve participants’ intended meanings. Quotations selected for inclusion in the manuscript were translated into English by a professional translator. The authors subsequently reviewed the English translations against the original Turkish statements to ensure semantic accuracy and consistency with participants’ intended meanings. Back-translation was not performed.

As a final validation step, the developed thematic structure was shared with selected participants to assess whether the interpretations adequately reflected their experiences; details of this member-checking process are provided under the Trustworthiness subsection. To ensure transparency and traceability, quotations were attributed to their respective focus groups using identifiers ranging from FG1 to FG11. Each identifier corresponds to a distinct focus group discussion, and the stakeholder category is provided alongside each quotation. As individual speakers were not systematically identified in the original transcripts, quotations were attributed at the focus group level rather than assigned retrospective individual participant identifiers.

### 2.6. Trustworthiness

Lincoln and Guba’s criteria of credibility, transferability, dependability, and confirmability were considered to enhance the trustworthiness of the study [18]. Credibility was supported through independent analysis by two researchers, external expert review, and member checking. Following the independent analysis and comparison of interpretations described above, the resulting thematic structure was reviewed by an academic expert who was not part of the research team and who had expertise in qualitative and phenomenological research.

Member checking was conducted with 16 participants, including 12 faculty members, two students, and two healthcare professionals. The two students and two healthcare professionals participated in member checking on a voluntary basis. Participants confirmed that the findings reflected their experiences, and their feedback did not necessitate any changes to the thematic structure.

Dependability and confirmability were supported by maintaining a close connection between the interpretations and the original data throughout the analytic process. When interpretations differed, the researchers returned to the transcripts and audio recordings before reaching agreement. Representative verbatim quotations from students, faculty members, and healthcare professionals were included in the findings to demonstrate the connection between participants’ accounts and the resulting interpretations. Transferability was facilitated by providing detailed information about the study setting, participant groups, sampling criteria, educational context, and data collection procedures, enabling readers to assess the applicability of the findings to other contexts.

### 2.7. Ethical Considerations

Ethical approval for the study was obtained from the Mardin Artuklu University Non-Interventional Clinical Research Ethics Committee (Decision No. 2023/5-3; 3 May 2023). Before the focus group interviews, participants were informed about the purpose of the study, the interview procedures, the voluntary nature of participation, and the confidentiality of the data. Written informed consent was obtained from all participants, and separate permission was obtained for audio recording. Participants were informed that they could withdraw from the study at any time without any consequences. The study was conducted in accordance with the principles of the Declaration of Helsinki.

## 3. Results

### 3.1. Participant Characteristics

A total of 48 participants took part in the study, including 20 senior nursing and midwifery students, 12 faculty members, and 16 healthcare professionals (eight nurses and eight midwives). The mean age of nursing students was 23.1 years, while that of midwifery students was 23.0 years. Among healthcare professionals, the mean age was 33.13 years for nurses and 34.63 years for midwives. Nurses and midwives were actively working in clinical units, including intensive care settings. All nurses held bachelor’s degrees. Among midwives (*n* = 8), five (62.5%) held bachelor’s degrees, two (25.0%) held associate degrees, and one (12.5%) held a master’s degree. The mean weekly working time was 43.71 h among nurses and 48.25 h among midwives. Among faculty members, 50% of those in the Nursing Department and all faculty members in the Midwifery Department were assistant professors. The mean duration of employment at the university was 15.93 years among nursing faculty members and 11.75 years among midwifery faculty members.

### 3.2. Overview of Themes

Analysis of the focus group data revealed four interconnected themes describing participants’ experiences of educational disruption during the COVID-19 pandemic and the 2023 earthquakes: (1) disrupted learning and unequal participation, (2) loss of experiential and clinical learning, (3) from educational disruption to professional competence concerns, and (4) reconsidering educational strategies during crises. Although students, faculty members, and healthcare professionals experienced these disruptions from different positions within the educational and clinical environments, their accounts converged most clearly around concerns regarding the consequences of disrupted clinical learning for professional competence. The themes and associated subthemes are presented in Table 2.

Although the four themes reflect experiences across both crises, the nature and duration of educational disruptions differed between the COVID-19 pandemic and the February 2023 earthquakes. Participants’ accounts of prolonged limitations in face-to-face learning and clinical experience primarily concerned the pandemic period, whereas their accounts of the post-earthquake period focused more specifically on changes in theoretical course delivery and the transition to hybrid education. These temporal distinctions are considered in the presentation of the findings below.

### 3.3. Theme 1: Disrupted Learning and Unequal Participation

#### 3.3.1. Difficulties in Online Learning

Students and faculty members described difficulties in understanding and delivering course content during online education. Students frequently compared online learning unfavorably with face-to-face education, particularly in relation to concentration and comprehension. One student explained:

“I had trouble understanding. I could understand better when it was face to face…” (FG2, Midwifery students.)

Faculty members similarly described difficulties in sustaining interaction and attention when teaching through a screen. One faculty member stated:

“I definitely had hard times… I had an adaptation problem… Speaking only to a machine in a very cold environment made me very tired… in the online system, you feel the need to take a break after 20 min.” (FG3, Nursing faculty.)

#### 3.3.2. Technological and Access Barriers

Technological access emerged as another important barrier. Students living in rural areas described unstable internet connections and the financial burden of internet access, while faculty members reported limitations in the capacity of the online learning system. One student noted, “Living in a mountain village, the network is already bad… internet package problems…” (FG8, Nursing students). A faculty member similarly described how the system became unstable when multiple students attempted to participate simultaneously: “You open a microphone and the system crashed. Considering that 90 microphones were opened, the system could not handle it.” (FG3, Nursing faculty). Faculty members also emphasized that access to technology was not equally distributed, noting that not every student had reliable internet access at home.

#### 3.3.3. Transition to Hybrid Education

Following the February 2023 earthquakes, the transition from distance education to hybrid delivery introduced additional challenges for students and faculty members. At the study institution, theoretical courses were delivered remotely until April 2023 and subsequently through a hybrid approach, while clinical and practical training continued face-to-face. Within this educational arrangement, some students described difficulties reorganizing their daily lives between home, university, dormitory accommodation, and clinical placements. One student stated:

“I think hybrid education disrupts our order. I come and stay in the dormitory and go home, but after I go home, I do not want to come here. Internship is torture for me…” (FG2, Midwifery students.)

Faculty members also questioned whether institutional conditions were adequately prepared for hybrid education, with one participant stating, “We are not a school suitable for the hybrid system… In fact, many schools in Turkey are not suitable for this.” (FG3, Nursing faculty.)

#### 3.3.4. Attendance and Motivation

Low attendance further complicated online teaching. Faculty members reported that the absence of compulsory live attendance reduced student participation and made it difficult to maintain engagement during lessons.

Faculty members additionally described reduced motivation during prolonged periods of online education and successive crisis-related disruptions. Teaching through a screen to a limited number of visibly engaged students was described as demotivating, and some faculty members expressed feelings of professional dissatisfaction and a reduced sense of professional value.

“During this period, I had a motivation problem. I was not motivated because student participation was very low, and we could not ensure their attendance. As a result, my motivation was zero, and I had no desire to teach because there was no one on the receiving end.” (FG1, Nursing faculty.)

#### 3.3.5. Domestic and Family Responsibilities

Home environments also shaped participation in online education. Both students and faculty members described how domestic responsibilities competed with academic work, particularly during periods when education was conducted from home. One student explained:

“When we were at school… we focused, but when we were at home… suddenly something came up, my mother said you can watch it later…” (FG4, Midwifery students.)

Faculty members similarly referred to childcare and household responsibilities as barriers to maintaining a regular working routine. One faculty member stated, “There were children at home or there was a babysitter” (FG3, Nursing faculty), while another described how gender roles confined them to the domestic environment and distanced them from their usual working lives. These accounts highlighted how domestic responsibilities and competing family demands affected participants’ ability to engage consistently in online education.

“I feel free when I leave the house. At home, I experience role conflict.” (FG3, Nursing faculty.)

Overall, participants’ accounts indicated that engagement with online and hybrid education was shaped not only by the mode of delivery itself but also by differences in technological access, home and family circumstances, changing educational routines, and opportunities for active participation.

### 3.4. Theme 2: Loss of Experiential and Clinical Learning

Participants described particularly marked disruptions to the experiential components of nursing and midwifery education during the COVID-19 pandemic when face-to-face educational activities were interrupted for an extended period.

Students and faculty members reported limited opportunities for laboratory practice, hands-on skill development, and direct patient care during this period of online education. For students, these restrictions created a perceived gap between theoretical learning and the practical experiences required to develop clinical skills. In contrast, clinical and practical training at the study institution continued face-to-face following the February 2023 earthquakes. Thus, the experiences of interrupted clinical learning described in this theme primarily relate to the pandemic period.

#### 3.4.1. Inadequate Practical Training

One student described the extent of these perceived deficiencies:

“It seems that 50% of our class did not have the opportunity to assist with a birth… they could not even measure blood pressure properly…” (FG4, Midwifery students.)

Another student reflected on the limited opportunities for clinical practice during the pandemic:

“We were very weak in terms of practice… because of the risk of infection.” (FG9, Nursing students.)

Faculty members similarly emphasized the limitations of delivering practice-based education remotely. They described some nursing and midwifery skills as inherently dependent on direct demonstration and hands-on experience. As one faculty member stated:

“Practical lessons could not be done remotely. You could not teach students how to administer injections remotely…” (FG3, Nursing faculty.)

Another faculty member emphasized the importance of patient contact for connecting theoretical education with clinical learning:

“It is not possible for us to provide theoretical training without seeing patients.” (FG11, Midwifery faculty.)

#### 3.4.2. Lack of Laboratory Experience

The absence of laboratory-based learning further restricted students’ opportunities to translate theoretical knowledge into practical skills before entering clinical environments. Students distinguished between understanding content presented during online courses and having opportunities to perform the corresponding procedures. One student explained:

“My teacher explained the gynecology course very well… but I could not practice on the models…” (FG2, Midwifery students.)

#### 3.4.3. Challenges in Patient Interaction

Direct encounters with patients further exposed students to situations in which they felt inadequately prepared. Students described uncertainty not only about performing clinical procedures but also about communicating with and responding to patients. One student stated:

“I feel unconfident in front of patients. We have not seen enough practice.” (FG8, Nursing students.)

Another explained:

“Sometimes I don’t know how to respond to the patient.” (FG2, Midwifery students.)

#### 3.4.4. Clinical Responses and Labeling by Healthcare Professionals

Returning to clinical environments after prolonged disruption presented another challenge. Students described entering clinical placements with limited previous hands-on experience and reported that these perceived skill gaps became visible during interactions with healthcare professionals. Some encountered critical or dismissive responses when they were unable to perform procedures expected of students at their stage of education. One student described being labeled according to the circumstances under which they had received their education:

“We were treated like you do not know… what kind of students are you, pandemic students, corona students, especially from the nurses! We were made fun of even when we could not inject. This wore us out a lot.” (FG9, Nursing students.)

Another student described the difficulty of entering clinical practice for the first time at a relatively advanced stage of the program:

“We went on our first internship in the third year because we took the practical courses remotely during the pandemic. ‘You are in the third year of the internship, why don’t you know?’… They found us very strange, so we felt very incomplete…” (FG2, Midwifery students.)

Faculty members also observed this labeling in clinical environments, reporting that students were sometimes characterized by healthcare professionals as “online students” who lacked expected knowledge and skills.

#### 3.4.5. Fear of Infection

Students also described fear of infection when clinical practice resumed during the pandemic. Concern about contracting COVID-19 and transmitting it to family members affected how some students approached patient contact. One student recalled:

“We were always wearing masks, I was constantly washing my hands, I was very scared if my family got infected… I was always watching from a distance…” (FG2, Midwifery students.)

Taken together, participants’ accounts suggest that pandemic-related disruptions to experiential learning extended beyond the temporary loss of laboratory and clinical training opportunities. Students described returning to clinical environments with limited opportunities to rehearse practical skills, develop confidence in patient interactions, and become familiar with the expectations of clinical practice. These experiences formed the context in which broader concerns about professional competence and preparedness for practice emerged across stakeholder groups. Importantly, these findings reflect perceived consequences of disrupted clinical education during the pandemic rather than an equivalent interruption of clinical training following the February 2023 earthquakes.

### 3.5. Theme 3: From Educational Disruption to Professional Competence Concerns

Concerns about professional competence emerged across all three stakeholder groups and represented one of the strongest areas of convergence in the data. These concerns were primarily associated with participants’ experiences of interrupted practical and clinical education during the COVID-19 pandemic. Students linked their limited opportunities for hands-on learning to uncertainty about whether they had developed the skills expected of them as they approached graduation. These accounts reflected perceived preparedness for professional practice rather than objectively assessed clinical competence. Although the February 2023 earthquakes introduced further changes in educational delivery, clinical and practical training at the study institution continued face-to-face during the post-earthquake period.

#### 3.5.1. Perceived Clinical Skill Deficits

One student questioned how professional competence could be developed in the absence of sufficient practice:

“…how are we going to learn this profession? It ended without practice… it created many shortcomings for us.” (FG9, Nursing students.)

#### 3.5.2. Reduced Confidence in Patient Care

Students also associated their limited practical experience with reduced confidence when providing patient care. Some described feeling anxious during their initial clinical procedures and becoming more confident as they gained hands-on experience. One student recalled:

“When I performed my first injection, my hands were shaking… As I practiced and repeated it, the fear gradually decreased.” (FG9, Nursing students.)

These accounts suggested that opportunities for repeated clinical practice were important not only for developing practical skills but also for building students’ confidence in patient care.

#### 3.5.3. Concerns About Readiness for Professional Practice

For some students, the proximity of graduation and employment made these concerns particularly immediate. A midwifery student stated:

“We will be assigned in a month, but I can’t sew an episiotomy yet.” (FG4, Midwifery students.)

Faculty members expressed similar concerns from an educational perspective. They perceived substantial gaps in the clinical experiences of students approaching graduation and questioned whether sufficient opportunities remained to compensate for what had been missed during the disrupted periods of education. One faculty member explained:

“This year, there is a huge gap in the midwives and nurses who will graduate… the clinic is very lacking… students will graduate without touching a patient and this worries me a lot. We really see the reflections of this in the clinic…” (FG1, Nursing faculty.)

Another faculty member expressed concern that these gaps could extend beyond graduation and affect the quality of future professional practice:

“The quality of service provided by future nurses will decline.” (FG1, Nursing faculty.)

#### 3.5.4. Healthcare Professionals’ Observations of Perceived Competence Gaps

Healthcare professionals provided a complementary clinical perspective on perceived gaps in preparedness for practice. Drawing on their experiences in clinical settings, they described difficulties involving students and newly entering healthcare staff. Their accounts included observations concerning the transition from education to clinical responsibilities, although these observations were not systematically linked to individual students participating in the focus groups. Accordingly, these findings represent healthcare professionals’ perceptions rather than objective assessments of clinical competence. One healthcare professional described difficulties assigning clinical responsibilities to newly entering staff:

“I could only put the ones who came before on duty in a month at most, with the new friends who came… The Intensive Care Unit did not accept the new ones in any way. They could not learn… how would I hand over a patient to them?” (FG5, Nurses.)

Another healthcare professional contrasted the clinical orientation needs of previous students with those whose education had been disrupted:

“The old students were just learning where the materials were, but the new students will learn how to give injections.” (FG10, Nurses.)

A midwife similarly emphasized that students were expected to demonstrate basic clinical assessment knowledge, even when they had not yet developed proficiency in performing clinical examinations:

“I do not necessarily expect a student to tell me how many centimeters dilated a patient is. That is something gained through practice. However, I expect them to know what should be assessed during an examination—dilatation, effacement, and fetal station—even if they cannot perform the examination or give me the correct answer.” (FG7, Midwife.)

Across the three stakeholder groups, concerns about professional competence emerged from different positions along the educational–clinical continuum. Students described uncertainty about their own preparedness, faculty members expressed concerns about the clinical experiences of students approaching graduation, and healthcare professionals reported perceived difficulties among students or newly entering staff in clinical settings. Although these perspectives reflected related concerns about preparedness for practice, they were not necessarily based on observations of the same individuals or cohorts. Therefore, the convergence of stakeholder perspectives should be interpreted as reflecting shared concerns about professional preparedness rather than demonstrating objectively measured competence deficits or a causal relationship between educational disruption and clinical performance.

### 3.6. Theme 4: Reconsidering Educational Strategies During Crises

#### 3.6.1. Difficulties in Assessment and Evaluation

Assessment was another area that participants believed required reconsideration. Students and faculty members questioned whether online examinations accurately reflected students’ knowledge and performance. One student stated:

“I don’t think online exams can differentiate between those who worked and those who didn’t… if my friend could study overnight, we would get the same score.” (FG2, Midwifery students.)

A faculty member similarly described discrepancies between students’ online examination scores and their demonstrated knowledge in clinical settings:

“There was a lack of confidence in exams… when the exam was online, some students received 93. That student… in the internship… could not answer even the simplest question…” (FG11, Midwifery faculty.)

#### 3.6.2. Development of Digital Skills

Despite these difficulties, participants also identified competencies gained during the transition to technology-supported education. Students and faculty members reported improvements in their ability to use digital tools. One student stated, “I learned how to use Word, and PowerPoint, and prepare surveys during this process” (FG2, Midwifery students), while a faculty member noted, “We have become much better at using technology” (FG3, Nursing faculty.)

#### 3.6.3. Divergent Views on Educational Decisions

Some participants considered the initial transition away from face-to-face education necessary under the circumstances. One healthcare professional reflected on the severity of the pandemic at the time:

“When the school opened… intensive care units were at their peak… it was the right decision… students might have been left without knowledge but… there was nothing left to do…” (FG5, Nurses.)

Another healthcare professional similarly stated:

“It was a wise decision for the students not to come…” (FG10, Nurses.)

Faculty members also emphasized that online education should not be regarded as inherently unsuitable for all forms of learning. One faculty member stated:

“Online education should not always be perceived as… disadvantageous. I think it can be beneficial, especially for courses that do not require practice.” (FG3, Nursing faculty.)

In contrast, other participants questioned whether the same approach should have been applied across different crises and educational contexts. Students distinguished between the circumstances of the pandemic and those following the earthquakes, suggesting that alternative arrangements could have been considered once the immediate emergency had passed. One student stated:

“I don’t think it was right for the earthquakes. It was a necessity at the time, but now a solution could be found. The pandemic was a little different.” (FG9, Nursing students.)

Faculty members also questioned the prioritization of educational closures during crises. One faculty member stated:

“Sacrificing education before anything else was not the right approach.” (FG11, Midwifery faculty.)

#### 3.6.4. Need for Technological and Financial Support

Participants emphasized that the continuity and accessibility of online and hybrid education during crises depended on adequate technological and financial resources. Students highlighted the need for support in accessing suitable devices and reliable internet connections, particularly for those facing financial or geographical barriers. Faculty members similarly emphasized the importance of strengthening institutional technological infrastructure to facilitate teaching and learning during periods of educational disruption.

#### 3.6.5. Maintaining Practical Education

Participants’ reflections frequently returned to the need to protect practice-based learning during crises. Considering possible educational arrangements during future crises, students suggested that clinical training could be organized through alternative local placements when access to the university or usual clinical training sites was restricted. One student proposed:

“When a disaster happens… It would be better if we worked in the hospitals in our own city… we would not be left behind.” (FG4, Midwifery students.)

Faculty members similarly argued that the practical requirements of nursing and midwifery education should be considered when crisis-related educational policies are developed. They also suggested the use of technology-supported alternatives, including virtual reality, to support practice-oriented learning when conventional clinical or laboratory education was disrupted.

#### 3.6.6. Stakeholder Involvement in Decision-Making

Faculty members emphasized that the specific requirements of nursing and midwifery education, particularly the importance of clinical and practical training, should be considered when educational policies are developed during crises. Some faculty members reported that their perspectives had not been adequately reflected in institutional decisions. One faculty member stated:

“Although we had faculty members representing us in the administration and explained our situation, our voices were not heard, or even when they were heard, nothing was put into practice.” (FG11, Midwifery faculty.)

Participants also emphasized the need for greater involvement of educators in future crisis-related decisions. As one faculty member suggested:

“They should involve faculty members in the decision-making process.” (FG1, Nursing faculty.)

## 4. Discussion

This study explored the experiences of nursing and midwifery students, faculty members, and healthcare professionals during successive educational disruptions associated with the COVID-19 pandemic and the February 2023 earthquakes in Türkiye. Four interconnected themes emerged, encompassing unequal participation, disrupted experiential learning, concerns about professional preparedness, and the reconsideration of educational strategies during crises. The findings highlight important differences between the two crises: the pandemic involved prolonged interruptions to face-to-face and practice-based education, whereas post-earthquake changes at the study institution primarily affected theoretical instruction, with clinical and practical training continuing face-to-face. Across stakeholder groups, participants emphasized the importance of preserving experiential learning while adapting educational delivery to crisis-specific circumstances.

Participants described multiple challenges in engaging with online and hybrid education, including difficulties in understanding course content, unstable internet connections, limited technological resources, reduced interaction, and difficulties adapting to changing modes of delivery. Similar challenges have been reported in nursing and midwifery education during the COVID-19 pandemic, particularly in relation to technological infrastructure, internet access, interaction, engagement, and the support required for effective online learning [6,11]. Importantly, the present findings indicate that participation was shaped not only by access to technology but also by the circumstances in which learning took place. Students described differences in internet availability and financial resources, while both students and faculty members referred to domestic and family responsibilities that competed with educational activities. Previous studies have similarly shown that access to equipment and reliable internet, financial resources, home-learning conditions, and work or family commitments can influence students’ experiences of online education [19,20,21]. These findings suggest that maintaining access to educational content does not necessarily ensure equal opportunities for meaningful participation when learners and educators experience markedly different technological and domestic conditions.

The challenges associated with technology-supported education were also evident following the 2023 earthquakes in Türkiye. In a nationwide study involving 1026 midwifery students conducted shortly after the earthquakes, attitudes toward e-learning were relatively low, while future anxiety was high, with more positive attitudes toward e-learning associated with lower levels of future anxiety [22]. These findings provide important contextual evidence that students’ experiences of technology-supported education following a disaster should be interpreted within the broader circumstances and uncertainties accompanying the crisis. The difficulties described during transitions between online, face-to-face, and hybrid education further highlight the importance of institutional preparedness for rapid changes in educational delivery. Previous evidence has shown that the abrupt transition to remote education during the COVID-19 pandemic required substantial adaptation of nursing curricula and teaching practices and created challenges for both students and educators [4]. In the present study, similar difficulties were described across successive crisis-related disruptions, but participants also emphasized that educational arrangements needed to respond to the specific circumstances of each crisis. This finding supports a shift from reactive changes in delivery toward proactive crisis preparedness, including adaptable curricula, technological capacity, and institutional plans that can be adjusted according to the nature and duration of disruption [23].

Reduced opportunities for experiential and clinical learning emerged as one of the most prominent concerns associated with the prolonged interruption of face-to-face education during the COVID-19 pandemic. Students described limited opportunities to practice clinical skills, use laboratory resources, interact with patients, and become familiar with clinical environments, while faculty members emphasized the difficulty of reproducing practice-based learning through remote education. These concerns should be interpreted in relation to the pandemic period, as clinical and practical training at the study institution continued face-to-face following the February 2023 earthquakes. Similar concerns have been reported across health professions education during the COVID-19 pandemic. In a qualitative study conducted with medical educators in Türkiye, the inability to maintain face-to-face clinical education was perceived to negatively affect the acquisition of clinical competencies and professional attitudes and values [24]. Likewise, qualitative research involving nursing students who continued clinical practice during the pandemic showed that direct participation in patient care, supervisory support, and opportunities to assume appropriate responsibility were important in shaping learning, whereas restricted participation and insufficient support could hinder learning and contribute to uncertainty about students’ own abilities [25]. More broadly, disruptions to clinical placements have been associated with reduced opportunities for skill development, confidence, and exposure to real-world practice [1].

The importance of clinical learning extends beyond opportunities to perform individual procedures. The development of clinical competence is supported by interactions among students, supervisors, clinical situations, and opportunities for feedback, reflection, and repeated practice [26]. Healthcare educators have similarly emphasized that effective supervision, feedback, and opportunities to participate in relevant clinical activities are important for students’ clinical competence and professional growth [27]. Evidence from medical education during the pandemic further illustrates the value of authentic clinical participation: students who assumed meaningful clinical responsibilities under supervision described opportunities to develop autonomy, communication, teamwork, decision-making, and professional growth [28]. Although this evidence comes from a different health profession and involved continued participation in clinical care rather than disrupted clinical education, it reinforces the broader educational importance of active participation in authentic clinical environments. From this perspective, the concerns expressed by participants in the present study may reflect not only reduced exposure to individual clinical procedures but also disruption of the broader experiential processes through which professional competence and confidence develop.

Concerns about professional competence and preparedness for practice were closely connected to these disruptions in experiential learning. Students described uncertainty about their ability to perform clinical procedures and communicate confidently with patients, while faculty members expressed concern about students approaching graduation with limited clinical experience. Previous research has similarly shown that disrupted clinical education may be accompanied by concerns about confidence, communication, adaptation to clinical environments, and readiness for practice among nursing students [1,12]. Importantly, these findings should not be interpreted as evidence that participants in the present study had objectively lower clinical competence, as competence was not directly assessed. Rather, they demonstrate how reduced opportunities for experiential learning were perceived by participants as influencing confidence and preparedness for professional practice. This distinction is important because perceived preparedness and objectively demonstrated competence are related but conceptually distinct outcomes. Readiness for professional practice is itself a multidimensional construct shaped by the integration of knowledge, clinical skills, professional judgement, interpersonal capabilities, and experiences within educational and workplace environments [2].

Perceptions of professional readiness following predominantly distance-based education have not, however, been uniformly negative. In a Turkish study of final-year nursing students who had received most of their education remotely, perceived professional readiness was reported to be relatively high, including in the domains of professional adaptation and competence [29]. This contrasting finding is important because it suggests that the perceived consequences of educational disruption may vary according to educational context, subsequent clinical learning opportunities, and the dimensions of readiness being assessed. It also supports a cautious interpretation of the present findings: participants’ concerns indicate perceived gaps in professional preparation but should not be generalized as evidence that students educated during periods of disruption necessarily enter practice with inadequate competence.

Another noteworthy finding was the use of labels such as “pandemic students” or “corona students” to describe students whose education had been disrupted by the pandemic. Although these expressions may reflect concerns about insufficient clinical preparation, they also raise questions about the potential stigmatization of students based on circumstances beyond their control. Previous research has demonstrated that nursing students’ sense of belonging in clinical settings is closely associated with their relationships with clinical staff, experiences of acceptance or exclusion, and opportunities for meaningful learning [30,31]. Furthermore, qualitative research has documented nursing students’ experiences of discrimination during clinical placements, highlighting the importance of inclusive and supportive clinical learning environments [32]. From this perspective, defining students primarily by the educational disruptions they experienced, rather than by their individual competencies and learning needs, may have implications for their clinical belongingness and developing professional identity, which is an important dimension of professional socialization [33]. Nevertheless, the present study did not directly examine whether these labels resulted in stigmatization, reduced confidence, or changes in professional identity. Therefore, these potential consequences should be interpreted cautiously. The findings nevertheless underscore the importance of avoiding generalized assumptions about crisis-affected student cohorts and providing supportive, individualized clinical learning opportunities.

The inclusion of three stakeholder groups provided complementary perspectives on concerns about clinical learning and professional preparedness. While students described uncertainty about their clinical abilities, faculty members expressed concerns about students approaching graduation, and healthcare professionals reported perceived difficulties among students and staff members who were new to clinical practice. Previous qualitative research involving clinical nurse educators and new graduate nurses similarly identified gaps in clinical and communication skills and lower confidence during the transition to practice following pandemic-affected undergraduate education [13]. Research involving newly graduated nurses working in emergency departments during the COVID-19 pandemic has also documented stress, work overload, and challenges associated with assuming professional responsibilities under crisis conditions [34]. Although these studies provide relevant context, the present findings reflect stakeholder perceptions rather than objectively assessed clinical competence. Furthermore, the observations reported by healthcare professionals were not systematically linked to individual student participants, and the study does not establish a causal relationship between educational disruption and clinical performance.

Previous qualitative evidence synthesis has examined nursing students’ experiences of teaching and learning during the COVID-19 pandemic, providing additional context for understanding the challenges associated with disrupted educational delivery [35]. The findings of the present study do not suggest that technology-supported education has no place in nursing and midwifery curricula. Online approaches can support selected theoretical content and, when appropriately designed, may contribute to the development of some practical skills; however, their effectiveness varies according to the skill, educational strategy, and technological modality used [5,6]. Technology-supported learning should therefore be considered part of a broader educational strategy rather than a direct substitute for supervised clinical experience.

Participants’ differing views regarding educational decisions during the COVID-19 pandemic and the 2023 earthquakes further suggest that crisis-related educational responses should be sensitive to the nature and circumstances of the disruption. While the transition to remote education during the pandemic was often perceived as necessary because of infection-related restrictions, participants questioned whether similar approaches were equally appropriate following the earthquakes. These findings do not constitute a direct comparison between the two crises; rather, they indicate that participants evaluated educational arrangements in relation to the specific conditions created by each disruption. This perspective is consistent with recent guidance on resilient health professions education, which emphasizes proactive preparedness, adaptable curricula, and context-responsive strategies rather than reliance on a single model of educational continuity [23]. Crisis preparedness in nursing and midwifery education may therefore require flexible plans that can be adapted according to the type, duration, geographical extent, and consequences of the disruption.

A recurring recommendation across participants’ accounts was that the practical requirements of nursing and midwifery education should be protected wherever feasible during periods of disruption. Suggestions such as arranging clinical placements in students’ local areas and using technology-supported alternatives, including virtual reality, reflected attempts to maintain experiential learning when conventional educational arrangements were not possible. Resilient health professions education similarly requires contingency strategies that preserve essential practice-based learning while adapting educational delivery to crisis conditions [23]. Simulation-based approaches may provide additional opportunities for students to practice clinical decision-making and skills when access to conventional clinical environments is restricted, with evidence indicating beneficial educational outcomes from scenario-based simulation in nursing education [36]. Virtual clinical learning may also support knowledge, skills, confidence, and learning experiences under appropriate conditions, although it should not be assumed to reproduce all dimensions of authentic clinical practice [37]. Accordingly, simulation and virtual approaches may be most useful as components of a broader contingency strategy that supplements, rather than automatically replaces, supervised clinical learning.

Participants also raised concerns about whether online assessments adequately reflected learning and preparedness for clinical practice, highlighting the need to align assessment strategies with cognitive, psychomotor, communication, and clinical decision-making competencies. At the same time, the development of digital skills was identified as a positive outcome of technology-supported education, demonstrating that crisis-related educational changes were not experienced exclusively as a loss. Participants’ calls for technological and financial support, together with greater involvement of students, educators, and healthcare professionals in educational decisions, further emphasize the importance of institutional preparedness and collaboration between educational institutions and clinical partners. Such collaboration may support the development of context-responsive contingency plans that preserve essential clinical learning opportunities while allowing flexibility in educational delivery during future crises [6,23].

### Strengths and Limitations

A key strength of this study is the inclusion of three stakeholder groups—nursing and midwifery students, faculty members, and healthcare professionals—which enabled educational disruption to be examined across the educational–clinical continuum. These complementary perspectives provided insight into how disruptions in experiential and clinical learning were experienced and interpreted by students and faculty members, alongside healthcare professionals’ observations drawn from their work with students and their broader clinical experiences. Another strength is the study context, in which participants had experienced successive changes in educational delivery associated with both the COVID-19 pandemic and the 2023 earthquakes in Türkiye. This provided an opportunity to explore educational experiences across different forms of crisis-related disruption rather than focusing on a single transition to emergency remote education. The use of independent analysis by two researchers, external expert review of the thematic structure, and member checking further supported the credibility of the findings.

Several limitations should be considered when interpreting the findings. First, the study was conducted at a single university and an affiliated training and research hospital in southeastern Türkiye. Although changes in educational delivery following the February 2023 earthquakes were implemented nationwide, some participating students came from earthquake-affected areas in southeastern and eastern Türkiye. Educational arrangements, institutional resources, clinical placement opportunities, and experiences of crisis may differ across institutions and settings; therefore, the transferability of the findings to other contexts should be considered in relation to these contextual characteristics. Additionally, information on participants’ sex was not collected, which limits the demographic characterization of the sample and precludes an examination of potential differences in experiences according to sex. Second, the researchers worked within the same institutional environment in which the study was conducted, and pre-existing academic or professional relationships with some participants were possible. Although independent analysis, comparison of interpretations, external expert review, and member checking were used to strengthen reflexivity and credibility, such familiarity may have influenced participants’ willingness to express critical or sensitive views and cannot be completely excluded. In addition, because data were collected through focus groups, participants’ accounts may have been influenced by group dynamics or by the presence and responses of other participants, particularly when discussing critical or sensitive experiences. Furthermore, although quotations were identified by stakeholder group and focus group number, individual speakers could not be reliably distinguished in the reported quotations, limiting the assessment of how widely particular perspectives were shared across participants. In addition, the study did not undertake a systematic comparison between nursing and midwifery participants. Third, the study explored participants’ experiences and perceptions of professional competence and preparedness for practice rather than objectively assessing clinical competence or patient-care outcomes. Accordingly, the findings should be interpreted as perceived consequences of educational disruption rather than evidence of objectively demonstrated competence deficits. Finally, the timing of data collection represents an important limitation. As the focus group interviews were conducted in May 2023, shortly after the February 2023 earthquakes, participants reflected on educational experiences spanning different phases of the COVID-19 pandemic and the post-earthquake period. Their accounts may have been influenced by recall and by the circumstances prevailing at the time of data collection. Furthermore, the absence of longitudinal follow-up prevents us from determining whether the concerns identified in 2023 persisted, diminished, or evolved as educational conditions changed. Accordingly, the findings should be interpreted as a context-specific account of participants’ experiences rather than as a representation of current educational conditions. Future multicenter studies involving diverse educational and clinical settings, and studies incorporating longitudinal or objective assessments of clinical competence, could further examine how crisis-related educational disruptions influence the transition from education to professional practice.

## 5. Conclusions

This study highlights how nursing and midwifery students, faculty members, and healthcare professionals experienced successive educational disruptions during the COVID-19 pandemic and the February 2023 earthquakes in Türkiye. The findings indicate that crisis-related educational arrangements should account for differences in the nature of disruptions and the requirements of theoretical and practice-based learning. Participants’ concerns about clinical competence and professional preparedness reflected their experiences and perceptions rather than objectively demonstrated deficits.

Future crisis preparedness in nursing and midwifery education should combine flexible theoretical instruction with strategies that preserve essential clinical learning. Context-sensitive planning, equitable access to educational resources, and collaboration between educational and clinical stakeholders may help institutions respond more effectively to different forms of disruption.

## Figures and Tables

**Table 1:** Educational delivery timeline for the 2019–2023 nursing and midwifery cohort.

Academic Semester	Educational Delivery Mode
Fall 2019–2020	Face-to-face education
Spring 2019–2020	Face-to-face until March 2020, followed by distance education
Fall 2020–2021	Predominantly distance education
Spring 2020–2021	Predominantly distance education
Fall 2021–2022	Predominantly distance education
Spring 2021–2022	Face-to-face education resumed
Fall 2022–2023	Face-to-face education
Spring 2022–2023	Distance education for theoretical courses until April 2023, followed by hybrid education; clinical and practical training continued face-to-face

Note: The timeline summarizes the predominant educational delivery arrangements. During the pandemic, educational practices could vary according to national decisions and institutional requirements.

**Table 2:** Themes, Subthemes, and Contributing Stakeholder Groups Derived from the Focus Group Data.

Theme	Subthemes	Contributing Stakeholder Groups
1. Disrupted Learning and Unequal Participation	Difficulties in online learning	Students; faculty members
	Technological and access barriers	Students; faculty members
	Transition to hybrid education	Students; faculty members
	Attendance and motivation	Students; faculty members
	Domestic and family responsibilities	Students; faculty members
2. Loss of Experiential and Clinical Learning	Inadequate practical training	Students; faculty members
	Lack of laboratory experience	Students
	Challenges in patient interaction	Students
	Clinical responses and labeling by healthcare professionals	Students; faculty members
	Fear of infection	Students
3. From Educational Disruption to Professional Competence Concerns	Perceived clinical skill deficits	Students; faculty members
	Reduced confidence in patient care	Students
	Concerns about readiness for professional practice	Students; faculty members
	Healthcare professionals’ observations of perceived competence gaps	Healthcare professionals
4. Reconsidering Educational Strategies During Crises	Difficulties in assessment and evaluation	Students; faculty members
	Development of digital skills	Students; faculty members
	Divergent views on educational decisions	Students; faculty members; healthcare professionals
	Need for technological and financial support	Students; faculty members
	Maintaining practical education	Students; faculty members
	Stakeholder involvement in decision-making	Faculty members

## Data Availability

The qualitative data generated and analyzed during the current study are not publicly available due to participant confidentiality but may be available from the corresponding author upon reasonable request, subject to ethical and institutional requirements.

## Supplementary Materials

The following supporting information can be downloaded at: https://www.mdpi.com/article/10.3390/healthcare14213772/s1, Supplementary File S1: Semi-structured focus group interview guides for students, faculty members, and healthcare professionals; Supplementary File S2: Completed Consolidated Criteria for Reporting Qualitative Research (COREQ) checklist.

## Abbreviations

COVID-19	Coronavirus Disease 2019
COREQ	Consolidated Criteria for Reporting Qualitative Research
CoHE	Council of Higher Education
LMS	Learning Management System
