# Supplementary material for: ‘How Are We Going to Learn This Profession?’: A Multi-Stakeholder Qualitative Study of Educational Disruption Across Pandemic and Earthquake Crises
Source: Healthcare (Basel). 2026 Nov 4;14(21):3772. doi: 10.3390/healthcare14213772 (PMC13762622; doi:10.3390/healthcare14213772)
Supplement: Supplementary file 1 [file healthcare-14-03772-s001.zip › Supplementary File_S1 (Focus Group Interview Guides).pdf]

Supplementary File S1.
Semi-Structured Focus Group Interview Guides

S1-A. Student Focus Group Interview Guide

1. How did the different teaching methods affect your theoretical knowledge and practical skills?

2. How did distance, hybrid, and face-to-face education affect your ability to provide patient care? How did you experience these different educational models?

3. What difficulties did you experience with distance, hybrid, and face-to-face education?

4. Which of the distance, hybrid, and face-to-face education models were you satisfied with, and why?

5. Looking back over the past four years, how would you evaluate the educational experiences of your classmates and the contributions of faculty members and clinical team members (nurses, midwives, and physicians)?

6. Different educational methods have been used, and future crises may occur. How do you think education could be improved in preparation for such situations?

7. What solutions would you suggest at the individual and institutional levels?

S1-B. Faculty Focus Group Interview Guide

1. How did using different teaching methods affect your experience of providing education?

2. What difficulties did you experience with distance, hybrid, and face-to-face education?

3. How did distance, hybrid, and face-to-face teaching affect your students' knowledge and skills?

4. In your view, how did these teaching methods affect students' patient care, knowledge, practical skills, and clinical decision-making abilities? How would you evaluate their clinical practice?

5. Which of the distance, hybrid, and face-to-face education models were you satisfied with, and why?

6. Looking back over the past four years, how would you evaluate the preparedness of recent graduates and the contributions of other faculty members and clinical team members (nurses, midwives, and physicians) to education?

7. Different educational methods have been used, and future crises may occur. How do you think education could be improved in preparation for such situations?

8. What solutions would you suggest at the individual and institutional levels?

S1-C. Nurse/Midwife Focus Group Interview Guide

1. During periods of the pandemic when students were absent from clinical settings, how did their absence affect you?

2. During periods of the pandemic when students participated in clinical practice, how would you evaluate their participation and clinical performance?

3. Did you experience any difficulties in clinical practice with students who had received distance, hybrid, or face-to-face education? Please explain.

4. In your opinion, how did these different educational methods affect students' clinical practice, knowledge and skills, patient care, and communication and interaction with you?

5. Looking back over the past four years, how would you evaluate recent graduates, faculty members, and clinical team members (nurses, midwives, and physicians) in relation to education and clinical practice?

6. Distance, hybrid, and face-to-face educational methods have been used, and future crises may occur. How do you think education could be improved in preparation for such situations?

7. What solutions would you suggest at the individual and institutional levels?
