# Supplementary material for: ‘How Are We Going to Learn This Profession?’: A Multi-Stakeholder Qualitative Study of Educational Disruption Across Pandemic and Earthquake Crises
Source: Healthcare (Basel). 2026 Nov 4;14(21):3772. doi: 10.3390/healthcare14213772 (PMC13762622; doi:10.3390/healthcare14213772)
Supplement: Supplementary file 1 [file healthcare-14-03772-s001.zip › Supplementary File S2 COREQ.pdf]

Supplementary File S2: COREQ checklist

Consolidated criteria for reporting qualitative studies (COREQ): 32-item checklist

Developed from:

Tong A, Sainsbury P, Craig J. Consolidated criteria for reporting qualitative research (COREQ): a 32-item checklist for interviews and focus groups. International Journal for Quality in Health Care. 2007. Volume 19, Number 6: pp. 349 – 357

Item No	Guide Questions/Description	Reported on Page #
Domain 1: Research team and reflexivity		
Personal Characteristics		
1. Interviewer/ facilitator	Which author/s conducted the interview or focus group?	pp. 7–9 (Sections 2.3–2.4)
2. Credentials	What were the researcher's credentials? E.g., PhD, MD	Not explicitly reported (credentials)
3. Occupation	What was their occupation at the time of the study?	p. 1 (author affiliations); pp. 7–8 (Section 2.3)
4. Gender	Was the researcher male or female?	Not reported (researcher gender)
5. Experience and training	What experience or training did the researcher have?	pp. 8–9 (Section 2.3); formal training not specified
Relationship with participants		
6. Relationship established	Was a relationship established prior to study commencement?	pp. 7–8; pp. 25–27 (Sections 2.3, 4.1)
7. Participant knowledge of the interviewer	What did the participants know about the researcher? e.g. personal goals, reasons for doing the research?	pp. 8–10 (Sections 2.4, 2.7); personal goals not specified
8. Interviewer characteristics	What characteristics were reported about the interviewer/facilitator? e.g. Bias, assumptions, reasons and interests in the research topic	pp. 7–8 (Section 2.3)
Domain 2: study design		
Theoretical framework		
9. Methodological orientation and Theory	What methodological orientation was stated to underpin the study? e.g. grounded theory, discourse analysis, ethnography, phenomenology, content analysis	pp. 5–6, 9–10 (Sections 2.1, 2.5)
Participant selection		

Item No	Guide Questions/Description	Reported on Page #
10. Sampling	How were participants selected? e.g., purposive, convenience, consecutive, snowball	pp. 6–7 (Section 2.2)
11. Method of approach	How were participants approached? e.g., face-to-face, telephone, mail, email	p. 8 (Section 2.2); invitation mode not specified
12. Sample size	How many participants were in the study?	p. 6-7; p. 11 (Sections 2.2, 3.1)
13. Non-participation Setting	How many people refused to participate or dropped out? Reasons?	p. 6-7 (Section 2.2; no refusals/withdrawals)
14. Setting of data collection	Where was the data collected? e.g., home, clinic, workplace	pp. 8–9 (Section 2.4)
15. Presence of nonparticipants	Was anyone else present besides the participants and researchers?	Not reported (presence of nonparticipants)
16. Description of sample	What are the important characteristics of the sample? e.g. demographic data, date	pp. 6-7, pp 11 (Sections 2.2, 3.1)
Data collection		
17. Interview guide	Were questions, prompts, and guides provided by the authors? Was it pilot tested?	pp. 8–9 (Section 2.4); Supplementary File S1; pilot testing not specified
18. Repeat interviews	Were repeat interviews carried out? If yes, how many?	Not explicitly reported (repeat interviews)
19. Audio/visual recording	Did the research use audio or visual recording to collect the data?	pp. 8–9 (Section 2.4)
20. Field notes	Were field notes made during and/or after the interview or focus group?	pp. 8–9 (Section 2.4)
21. Duration	What was the duration of the interviews or focus group?	pp. 8–9 (Section 2.4)
22. Data saturation	Was data saturation discussed?	pp. 8–9 (Section 2.4)
23. Transcripts returned	Were transcripts returned to participants for comment and/or correction?	Not reported (transcripts returned); member checking of findings on pp. 10
Domain 3: analysis and findings		
Data analysis		
24. Number of data coders	How many data coders coded the data?	pp. 9–10 (Section 2.5; two researchers)
25. Description of the coding tree	Did the authors provide a description of the coding tree?	pp. 9–10 (Section 2.5); pp. 12 (Table 2)

Item No	Guide Questions/Description	Reported on Page #
26. Derivation of themes	Were themes identified in advance or derived from the data?	pp. 9–10 (Section 2.5)
27. Software	What software, if applicable, was used to manage the data?	Not reported (software)
28. Participant checking	Did participants provide feedback on the findings?	pp. 10 (Section 2.6)
Reporting		
29. Quotations presented	Were participant quotations presented to illustrate the themes/findings? Was each quotation identified? e.g., participant number	pp. 12–20 (Section 3; focus-group labels)
30. Data and findings consistent	Was there consistency between the data presented and the findings?	pp. 12–20 (Section 3)
31. Clarity of major themes	Were major themes clearly presented in the findings?	pp. 11–12 (Section 3.2, Table 2)
32. Clarity of minor themes	Is there a description of diverse cases or a discussion of minor themes?	pp. 11–12 (Table 2); pp. 11–20 (Section 3)
